# Design and Fabrication of a BiCMOS Dielectric Sensor for Viscosity Measurements: A Possible Solution for Early Detection of COPD

**DOI:** 10.3390/bios8030078

**Published:** 2018-08-21

**Authors:** Pouya Soltani Zarrin, Farabi Ibne Jamal, Subhajit Guha, Jan Wessel, Dietmar Kissinger, Christian Wenger

**Affiliations:** 1 IHP, Im Technologiepark 25, 15236 Frankfurt/Oder, Germany; jamal@ihp-microelectronics.com (F.I.J.); guha.ihp2608@gmail.com (S.G.); wessel@ihp-microelectronics.com (J.W.); dkissinger@ihp-microelectronics.com (D.K.); wenger@ihp-microelectronics.com (C.W.); 2 Institute of High-Frequency and Semiconductor System Technologies, Technical University of Berlin, 10623 Berlin, Germany; 3 Brandenburg Medical School Theodor Fontane, 16816 Berlin, Germany

**Keywords:** Sputum–Mucin, dielectric measurements, CMOS viscosity sensor, radio frequency sensor, Viscometer, biosensors, precision diagnostics

## Abstract

The viscosity variation of sputum is a common symptom of the progression of Chronic Obstructive Pulmonary Disease (COPD). Since the hydration of the sputum defines its viscosity level, dielectric sensors could be used for the characterization of sputum samples collected from patients for early diagnosis of COPD. In this work, a CMOS-based dielectric sensor for the real-time monitoring of sputum viscosity was designed and fabricated. A proper packaging for the ESD-protection and short-circuit prevention of the sensor was developed. The performance evaluation results show that the radio frequency sensor is capable of measuring dielectric constant of biofluids with an accuracy of 4.17%. Integration of this sensor into a portable system will result in a hand-held device capable of measuring viscosity of sputum samples of COPD-patients for diagnostic purposes.

## 1. Introduction

Chronic Obstructive Pulmonary Disease (COPD) is one of the leading causes of death among developed countries [1]. Although studies suggest that the early diagnosis could effectively decrease the mortality rate of COPD, many patients go undiagnosed until the late stages of their disease [2]. Abnormal sputum, infection, and bronchial spasm are the three main syndromes of chronic bronchial disease. Sputum specimens contain saliva, serum transudate, and glycoproteins. Mucus glycoproteins (mucin) are good mediums for bacterial growth and are responsible for the viscous properties of mucus. As a result, characterization and rapid screening of the mucus could be used for early diagnosis of COPD [3]. Since the muco–protein content and the hydration of the sputum determine its viscosity level, monitoring the viscosity of the sputum collected from a patient could provide useful diagnostic information for the disease [3,4].

Recent advancements in the development of viscosity sensors for biofluids are discussed in [5]. A wide range of commercially available viscometers were designed based on piezoelectric sensors. For example, Microvisk (Microvisk Ltd., Oxfordshire, UK), a commercially available viscometer, analyzes glucose concentration in blood for Point-of-Care (POC) applications using Micro Electro Mechanical Systems (MEMS). Similarly, various research-oriented viscometers have been developed for blood analysis or coagulation monitoring using the MEMS technology [5,6,7]. The key principle of these piezoelectric-based viscosity sensors is based on the deflation of their piezo material-based cantilevers or beams when they are exposed to the viscous fluid. Deformation of these structures generates an electrical output depending on their readout mechanism. As a result, the generated electrical output indicates the viscosity of the fluid exposed to the sensor. Although MEMS-based sensors are one of the most well-established sensing technologies, some drawbacks restrict their application for viscosity measurements. The main disadvantage of these sensors is the resetting of their cantilevers back to the original position, causing calibration and accuracy issues. Moreover, the viscous nature of the sensing fluid causes a considerable amount of damping on the cantilever vibration, impairing the sensor’s function. Furthermore, there is a significant amount of coupling between the fluid flow and the cantilever vibration that influences the acquired results from the sensor. In addition, the existing amount of drift in these sensors questions their reliability for long term operations [7]. In a work by Cakmak et al., a MEMS-based viscosity sensor was developed on a Complementary Metal Oxide Semiconductor (CMOS) platform. Although miniaturization of the system reduced damping effects and fluid flow rate, no significant improvements in terms of sensor accuracy or drift were achieved [8]. As an alternative technology, a pressure-based viscometer was developed for measuring blood glucose levels [9]. The needle-type glucose sensor consisted of two hollow fibers in which the viscous fluid was pumped up with a constant rate of 5 μL/h. In this design, two pressure sensors were placed at both ends of the fibers and the pressure difference created by the flow determined the viscosity of the sample fluid. Apart from the innovative design of the sensor, its long response time (5 to 10 minutes), low resolution, and limited accuracy made its clinical application unacceptable [9]. In contrast, optical biosensors are known as a well-established modality capable of providing accurate results for clinical applications [10,11,12,13]. However, they suffer from a few disadvantages for POC applications such as high power consumption, high cost, large sample volume requirements, lack of portability, operation complexity, and incapability of label-free measurements. Alternatively, Kuenzi et al. designed and developed a magnetically actuated rotational microviscometer [14]. The generated viscous friction by the fluid on the rotating magnet affected its rotation speed and angular position which were recorded for viscosity measurements. Although the system was able to provide accurate results, its bulky size limited its use for real-world POC systems [14].

Silicon-based technologies such as CMOS provide countless advantages for POC and Internet-of-Things (IoT) applications such as miniaturization, portability, high accuracy, reliability, low cost, high noise immunity, low power consumption, and complete integration with Lab-On-a-Chip (LOC) [15]. Due to these advantages, CMOS-based dielectric sensors have been used in numerous biological applications such as microorganism detection and characterization, neuronal activity detection, dielectric spectroscopy for medical diagnosis, and disease detection [16,17,18]. The developed biosensors detect and characterize biological targets based on their intrinsic properties or biochemical reactions. These biological parameters include biomarkers, biomolecules, proteins, DNA, pathogenic organisms, hormones, medical analytes such as glucose, and medical parameters like blood pressure [17,18]. For example, CMOS-based electrochemical sensors have been designed and developed for DNA detection and characterization [19,20,21]. In these works, Interdigitated Capacitors (IDC) were used to determine the biochemical properties of DNA molecules. An IDC sensor is a parallel-plate capacitor whose electrodes are positioned horizontally to provide a single sided access to the Material-Under-Test (MUT). In other words, the dielectric constant (relative permittivity) of the MUT defines the capacity of the IDC [22]. IDC-based sensors have been used in various studies to detect the dielectric constant of organic fluids as well as characterization of biological cell suspensions [23,24]. It is noteworthy that the relative permittivity is a frequency-dependent complex number that represents the characteristics of a medium in an interaction with electromagnetic fields. The real part of the permittivity is known as the dielectric constant, which represents the amount of energy absorbed by the material from an electromagnetic field. For medical applications, measuring the real part of the permittivity is useful for medium characterization like estimating the glucose concentration or determining the ratios of specific mixtures. On the other hand, the imaginary part of the permittivity reflects the materials energy loss to an external electric field. For example, conductivity of the material (which is the feature used for detecting the ratio of dead sperms during semen analysis) is correlated to the energy loss [25]. Over recent decades, researchers have investigated the correlation between the dielectric properties of biological samples (e.g., blood) and specific diseases at microwave frequencies [26]. It has been shown that biomolecules and biocells existing in analyzed biological samples provide different dielectric characteristics for subjects with diseases compared to healthy ones [18]. The same concept is applicable for sputum samples collected from patients diagnosed with COPD, as previously mentioned. At different stages of COPD, the viscosity of the sputum varies due to its hydration change. Since the dielectric constant (permittivity) of the sputum is correlated to its hydration, it is possible to characterize the viscosity of the sputum by measuring its dielectric constant.

In spite of the aforementioned advancements in developing biosensors for various POC applications, a reliable technology for early diagnosis and monitoring of COPD is still missing. Considering the fact that the viscosity of the sputum sample collected from a COPD diagnosed patient is a reliable indicator of the disease presence, development of a portable viscometer capable of providing real-time measurements with high accuracy and resolution is significantly important. Therefore, the objective of this work was to design and develop a CMOS-based dielectric sensor for real-time viscosity characterization of controls such as ethanol, methanol, and isopropanol. This work was aimed to identify the shortcomings of our previous prototype and develop a newer generation of the dielectric sensor with a proper packaging and improved accuracy [27,28]. Future integration of this biosensor into a medical device can be used for early diagnosis of COPD through viscosity characterization of sputum. The working principle of the intended sensor is based on the dielectric measurement of the MUT using capacitance sensors mounted on a CMOS platform. The capacitance change of the sensor affects the free oscillation frequency of the LC resonant tank which would be used for the readout mechanism. The operating frequency of the sensor is chosen to be in the range of 10–30 GHz, where the permittivity of water is considerably high compared to other existing biomaterials in the sputum [17]. Due to the existing permittivity contrast in the mentioned frequency range, a better Signal-to-Noise Ratio (SNR) is expected. In addition, a high frequency sensor has generally a small-sized chip which significantly reduces the sample volume requirements. Furthermore, the mentioned frequency range provides the most accurate results for dielectric sensing applications considering the fact that the undesired parameter-dependent dispersion mechanism of the biological cells, which exists in low-frequency ranges, has negligible effects on the sensor functionality. On the other hand, based on the single Debye’s relaxation mechanism, the dielectric permittivity of water decreases after 17 GHz making extremely high frequency measurements inadequate for the intended application [17].

## 2. Materials and Methods

### 2.1. First Generation of the Sensor

A first prototype of the CMOS-based dielectric sensor for the detection of viscosity changes in sputum–mucin was presented previously [27,28]. The details of this prototype along with its modified version are presented below.

#### 2.1.1. Sensor Design and Operation Principle

**Figure 1:**
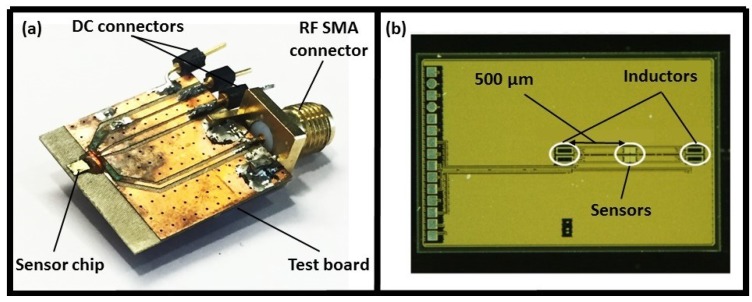
(**a**) First generation of the dielectric sensor, (**b**) sensor chip showing IDCs and inductors embedded in a CMOS oscillator [27,28].

A CMOS-based Radio Frequency (RF) dielectric sensor for the characterization of the sputum–mucin was designed and manufactured, as shown in Figure 1a. The sensor was fabricated through the standard 250 nm SiGe:C BiCMOS technology of IHP (IHP, Frankfurt/Oder, Germany) and operated at a frequency of 12 GHz. The working principle of the sensor was based on measuring the dielectric constant of liquid samples in order to characterize their viscosity. For this purpose, planar IDC sensors were coupled with inductors forming an LC resonant tank for the CMOS oscillator, as shown in Figure 1b. The capacitance of the IDC varied based on the dielectric constant of the MUT, leading to a change in the oscillation frequency. As a result, frequency changes of the oscillator indicated the viscosity variation of the MUT.

**Figure 2:**
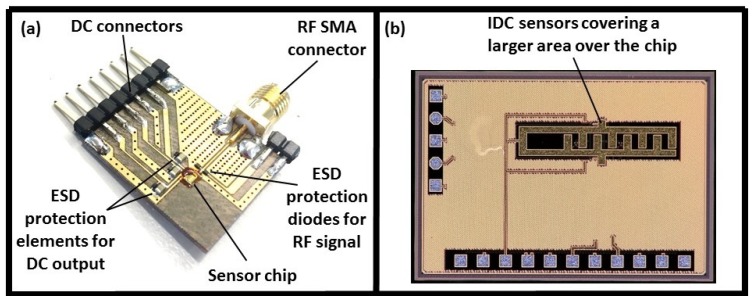
(**a**) ESD-protection elements mounted on the PCB, (**b**) modified chip with a larger sensing area coverage.

Although the developed sensor proved the practicality of the concept of viscosity characterization using dielectric sensors, its susceptibility to Electrostatic Discharges (ESD) made handling the system problematic. As a result, ESD-protection diodes (ESD9B, ON Semiconductor, CO, USA) and capacitors (0603 X5R:EIA, Murata Manufacturing Co., Nagaokakyo, Japan) were mounted on the Printed Circuit Board (PCB) in order to reduce the impact of ESD distortions on the sensor functionality, shown in Figure 2a. In order to increase the accuracy and sensitivity of the sensor, a new chip with a larger sensing area coverage (with wider IDC elements) was designed, as demonstrated in Figure 2b. The details of the circuit design is available in our previous work [27,28,29].

#### 2.1.2. Sensor Performance and Required Modifications

The sensor was initially calibrated using ethanol, methanol, acetone, and isopropanol with known permittivity and viscosity values. Following sensor calibration, two sets of experiments were performed to evaluate the sensor. First, two solution mixtures (glycerol–water and glycerol–ethanol) were characterized with varying water and ethanol contents in order to obtain different permittivity and viscosity values. Second, the sensor performance was validated by characterizing the permittivity of three different biological liquids including: human serum, human saliva, and sputum–mucin clot. The sensor provided an oscillation frequency change of 200 MHz for a change of 60% in the sputum–mucin viscosity. The details of the assessment methods and its results are available in [27,28].

The first prototype of the sensor provided promising results and proved the feasibility of the method for measuring the viscosity of biological samples using dielectric sensors. However, a few limitations associated with the system restricted its use in real-world applications. These issues are as follows:

1. Even though the RF output of the first-generation sensor was useful for conducting pilot experiments and evaluating the system during preliminary studies, its numerous drawbacks made it an unfavourable choice for out-of-the-lab applications. The main drawback of the RF output was the necessity of having costly and bulky spectrum analyzers for the signal acquisition which is an unrealistic intention for the development of POC devices. In addition, the RF signal is generally very sensitive to external distortions which causes a considerable amount of noise on the sensor outcome.
2. Due to the lack of an adequate packaging, the sensor was extremely susceptible to ESD-caused damages. As a consequence, handling the system during the wire bonding process, soldering of PCB elements, and running experiments were significantly inefficient and complicated. Moreover, the spreading of conductive liquids on the PCB surface, especially during the characterization of biological samples containing water, caused the sensor to short-circuit. Hence, a proper packaging for ESD protection and short-circuit prevention of the system was required.
3. Considering the inhomogeneous nature of biological liquids, including mucin and saliva, a series of sensing elements were required to increase the overall sensing ability of the system and improve its repeatability.
4. Since the sensor measures the dielectric constant of a sample, electrical features of the sample determine the sensor outcome rather than its mechanical features. For instance, increasing the concentration of ethanol in an ethanol–glycerol mixture decreases both the permittivity and the viscosity of the mixture. Conversely, increasing the concentration of water in a water-glycerol mixture increases the permittivity of the mixture while decreasing its viscosity. Thus, the measured permittivity for a given viscous sample is dependent on the constituents of that sample. In other words, it is only the permittivity of the solution which is detected by the sensor rather than its absolute viscosity values. This is due to the fact that there is no direct mathematical correlation between the viscosity and any electrical quantity. Consequently, different calibrations are required based on the intrinsic characteristics of the tested samples. This issue causes calibration complexity and makes the viscosity detection of unknown samples impractical. Therefore, a more reliable calibration and validation method is necessary to improve sensor outcome. For this purpose, the direct calibration of the sensor using a commercialized viscometer is recommended.

A second generation of the sensor prototype was designed and developed in order to address these shortcomings, as presented in the following section.

### 2.2. Second Generation of the Sensor

**Figure 3:**
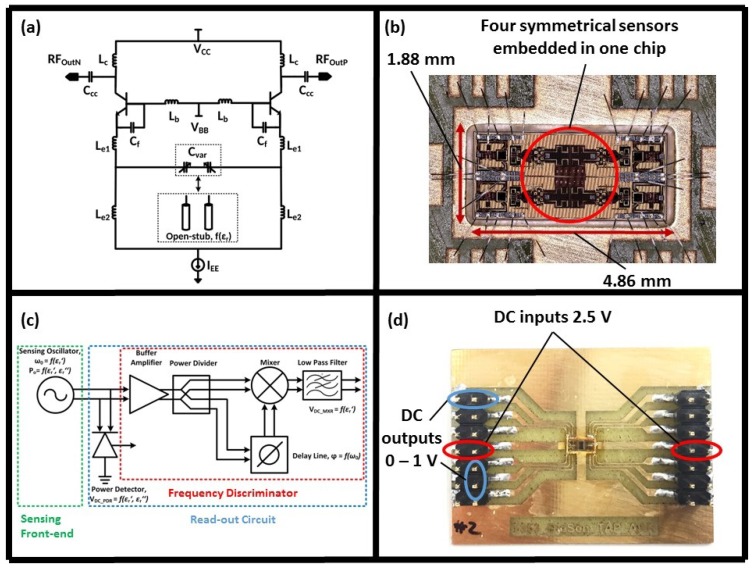
(**a**) Sensor oscillator circuit [30], (**b**) second-generation chip with a quadruple-sensor configuration, (**c**) integration of the DC readout circuit and the sensor on the CMOS platform [31], (**d**) test board of the second-generation dielectric sensor with DC inputs and outputs.

Functioning on the same sensing principle as the first prototype, a new version of the sensor for the detection of dielectric constant changes due to viscosity variations of sputum–mucin was developed, Figure 3. Capacitive elements were coupled with inductors to define the oscillation frequency of the resonator component. In this design, the capacitive elements are a pair of microstrip open-stubs with an electrical length below the quarter-wavelength of the sensor operation frequency, as shown in Figure 3a. The operation frequency of the sensor is in the range of 30 GHz, which results in a high SNR [17]. Similar to the previous sensor, the 250 nm SiGe:C BiCMOS technology of IHP was used to fabricate the sensor. The limitations of the first prototype were addressed in order to improve the sensor functionality.

#### 2.2.1. Sensor Design and Functionality

As shown in Figure 3b, the second generation of the sensor with a 9.2 mm${}^{2}$ chip size was designed and fabricated. The larger size of the chip, compared to previous generations, is due to two reasons: quadruple-sensor design for the detection of inhomogeneous samples, Figure 3b—the full integration of the complementary readout circuit and the sensor on the CMOS platform, Figure 3c.

Unequal dispersion of inhomogeneous fluids over the sensing area of the first generation of the sensor led to poor repeatability. In order to address this issue, four analogous sensors were integrated in a quadruple design to increase the sensing area in contact with MUT and to reduce sample dispersion effects on the sensor measurements, as shown in Figure 3b. Despite having four sensors, the chip consumes 80 mW power which makes it an appropriate technology for POC devices.

The readout mechanism for the new generation of the sensor was modified to eliminate noise caused by the RF output. As demonstrated in Figure 3c, a frequency discriminator was implemented into the sensor readout circuit to convert the RF information into a DC output. Subsequently, a power detector was used to extract the output power. Therefore, DC signals corresponding to the sensor oscillation frequency were generated as the system output. Figure 3d illustrates the inputs and outputs for all four sensors. Two DC inputs of 2.5 V are required for the system power source. On the other hand, each sensor provides two DC outputs in a range of 0–1 V for the real and imaginary parts of the measured permittivity, as shown in Figure 3d. Further details regarding the circuit design are available in our previous work [30,31]. Using DC inputs and outputs, the amount of perturbation on the system was diminished and the sensor performance was significantly improved. In addition, the low power consumption of sensors and ease of handling DC signals made the integration of the whole system into a compact portable device possible. The packaging process of the system is described in the following section.

#### 2.2.2. System Packaging

**Figure 4:**
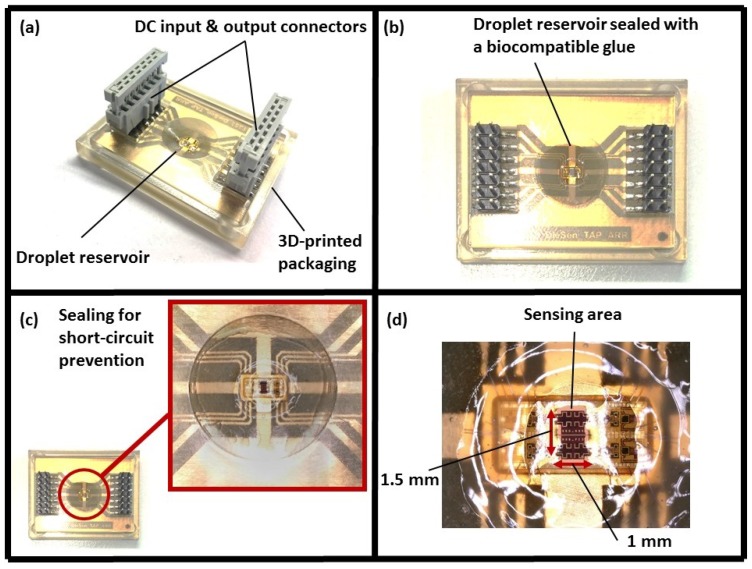
(**a**) The sensor packaging, (**b**) droplet reservoir, (**c**) sealing of the reservoir for prevention of liquids spreading, (**d**) remaining sensing area after the sealing step.

Figure 4a illustrates the packaging of the sensor, which was fabricated using a 3D printer at IHP (Keyence Agilista-3200W, Keyence Co., Osaka, Japan). A droplet reservoir was considered on the packaging design for holding liquid samples, as shown in Figure 4b. A medical grade biocompatible and electronics-friendly glue (Loctite M-21HP, Henkel AG & Company, KGaA, Dusseldorf, Germany) was used to seal the droplet reservoir thoroughly, as shown in Figure 4c. As a result, the short circuit of the sensor due to the spreading of the conductive liquids on the chip surface was prevented. Additionally, the bond wires were covered by a non-conductive biocompatible glue (TNP0400, Kyocera Corporation, Kyoto, Japan) to protect them during assembling the packaging and testing samples. Figure 4d illustrates the remaining sensing area for sample measurements after the gluing process.

The ESD damages caused by the direct contact of the operators’ hands with the test board during conducting experiments were minimized using the developed packaging. Moreover, the packaging simplified the cleaning process of the chip after conducting measurements, since using conductive liquids such as water for cleaning the sensing area was possible. Therefore, the system has become more user-friendly to handle and conduct experiments.

#### 2.2.3. Experimental Setup

**Figure 5:**
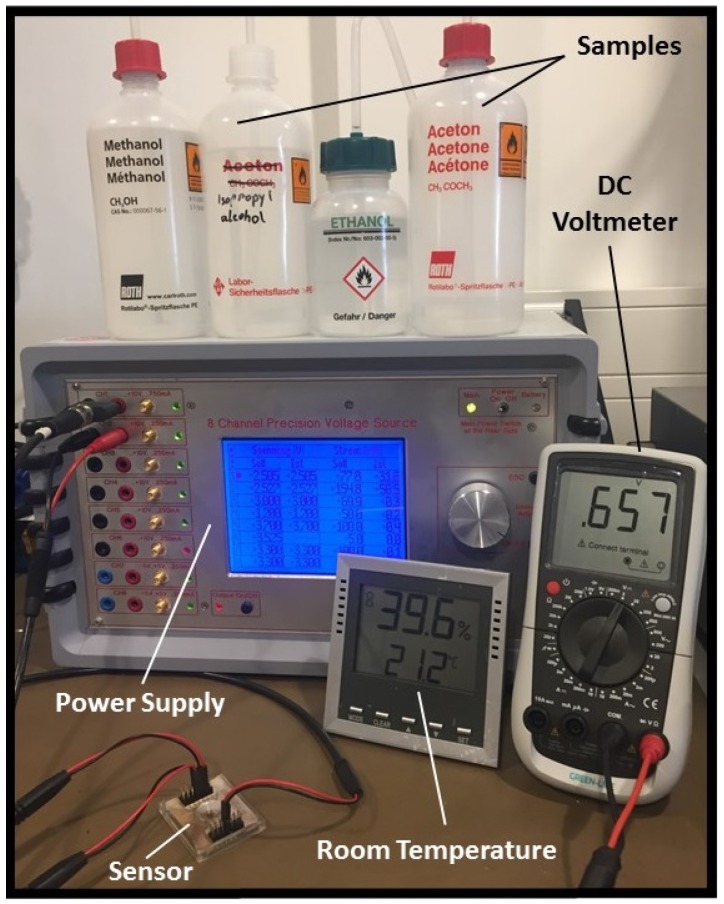
Measurement setup for the sensor calibration and validation experiments.

Since the dielectric constant of materials is highly temperature dependent, all experiments for the sensor calibration and assessments were performed in a lab with a sustained temperature of 21 ${}^{\circ }$C, as shown in Figure 5. Considering the frequency dispersive behavior of materials, Debye-based relaxation equations were used to calculate the dielectric constants of the tested materials at 21 ${}^{\circ }$C and 30 GHz operating frequency of the sensor, as presented in Table 1 [32,33,34,35].

## 3. Results and Discussions

### 3.1. Calibration

The initial measurements of the sensor with no MUT features the dielectric characteristics of the surrounding air. As a result, the dielectric constants of air, isopropanol, ethanol, and acetone were used to calibrate the sensor for dielectric measurements.

**Figure 6:**
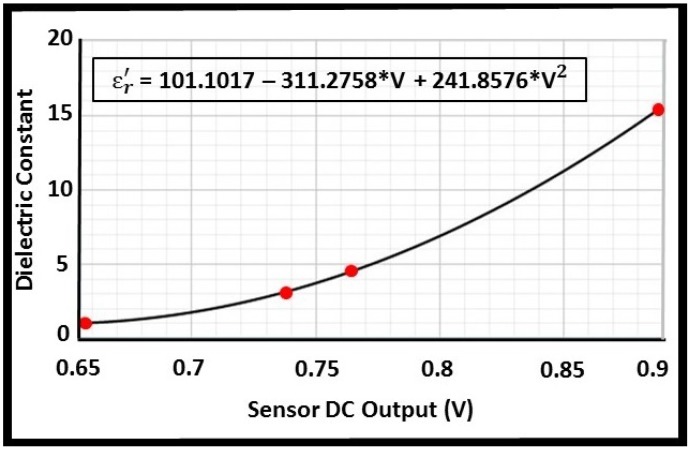
The fitted calibration line using the quadratic regression method. In the equation, *V* represents the corresponding DC output of the sensor in volt and $\varepsilon_{r}^{\prime }$ is the dielectric constant of the MUT.

The quadratic regression method was used on the obtained results to establish a relationship between the sensor output and the dielectric constant of the corresponding material. As illustrated in Figure 6, the calibration line was fitted based on the calculated coefficients of the quadratic regression expression.

### 3.2. Performance Assessment

Following sensor calibration, experiments were performed on methanol to evaluate the sensor efficacy. As presented in Table 2, the experiments were conducted in triplicate and the dielectric constant of methanol was calculated using the calibration equation presented in Figure 6. Based on the obtained results, the following performance measures were calculated:

- Accuracy: calculated as the difference between the actual and the measured value divided by the actual value (relative error). The total error of all three sets of measurements is reported.
- Repeatability: presented as the maximum standard deviation of the errors observed during three experiments.
- Hysteresis: calculated as the difference between the sensor initial measurements before and after performing an experiment. The highest value of all trials is reported.
- Drift: the sensor output with no MUT was recorded from the initiation of the system for a time period of 10 minutes. Drift was calculated as the difference between the lowest and the highest dielectric constant value measured during the first and last 10 seconds.
- Noise: calculated as the difference between the lowest and the highest dielectric constant value acquired in a 10 second data set with no MUT.

**Figure 7:**
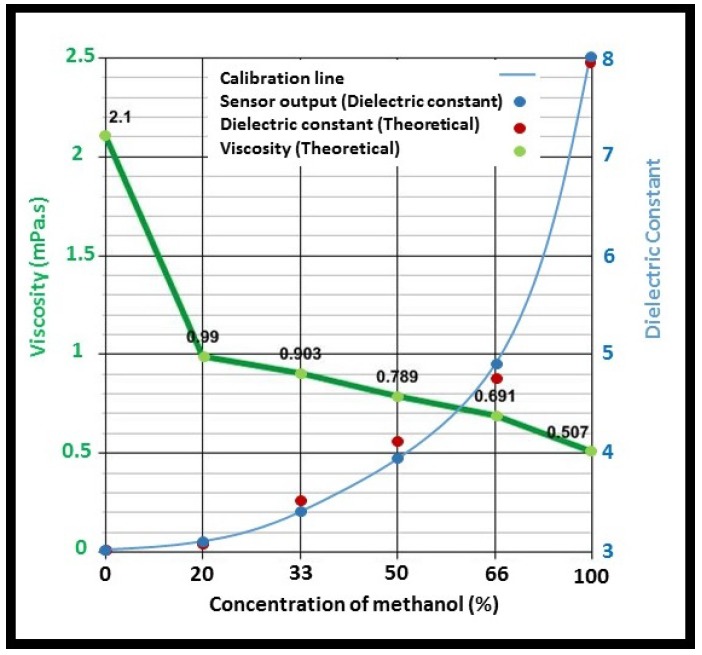
Viscosity and permittivity variation of the isopropanol-methanol mixture with respect to methanol content.

The functionality of the sensor to detect viscosity variation was evaluated by the mixture characterization method. Six mixtures of methanol-isopropanol, ranging from 0% methanol to 100% methanol (in volumes), were prepared. The effective permittivity and viscosity values of the mixtures at the working frequency of the sensor were calculated using mixture theories [28,30,36]. The theoretical values of the mixture permittivity and viscosity are plotted in Figure 7. The sensor results of the dielectric constant of the mixtures were calculated based on the sensor calibration and illustrated in Figure 7.

The results of the experimental evaluation are presented in Table 3. The sensor is capable of measuring the dielectric constant of the MUT with an accuracy of 4.17% and a repeatability of 5.36%. Therefore, the results of the second-generation sensor are more reliable compared to the first prototype. Furthermore, a hysteresis value of 2 mV was observed to have a negligible effect on the sensor measurements.

The issues related to the susceptibility of the RF signal to external distortions were addressed and a more stable system with DC readout mechanism was developed. Therefore, the level of noise existing in the system has been remarkably reduced (1 mV) compared to the first-generation sensor. In addition, the sensor shows a low amount of drift (5 mV) which results in a calibration consistency during long term measurements. The low drift and hysteresis characteristics of the sensor make it a more reliable technology for real-world applications.

The short-circuiting issue of the sensor was addressed using the sensor packaging. The droplet reservoir of the packaging was thoroughly sealed in order to prevent the spreading of the conductive samples on the board during experiments. Furthermore, the packaging prevented the direct contact of operators’ hands with the board for ESD-protection.

Quadruple design of the chip improved the overall sensing ability of the system and therefore, more repeatable and consistent results were achieved. Further investigation to evaluate the sensor performance during the characterization of inhomogeneous fluids is required.

Figure 7 shows the variation of the viscosity and dielectric constant of the isopropanol-methanol mixture with respect to the concentration of methanol. It is shown that with the addition of methanol, the viscosity of the resultant mixture decreases due to the low viscosity of methanol. Conversely, the permittivity of the solution increases considering the high permittivity of methanol. As a result, the sensor is able to detect the viscosity variation of the solution based on its permittivity changes. However, it should be noted that it is required to know the dielectric characteristics of the mixture’s constituents to be able to detect its viscosity variation. Estimation of absolute viscosity values using a dielectric sensor is not feasible since there is no direct mathematical correlation between viscosity and an electrical quantity.

Although capabilities of the system were evaluated through the characterization of control liquids with known dielectric constant values, a direct viscosity calibration and validation method is still missing. Our team is currently investigating this approach using a commercialized viscometer (m-VROC, RheoSense Inc., San Ramon, CA, USA) to correlate the absolute viscosity values of saliva and sputum samples to the sensor results. However, the complexity of viscosity measurements of non-Newtonian fluids make it a challenging approach. These investigations are still ongoing.

The second generation of the sensor was able to address the limitations of the previous design. The drawbacks related to the RF output of the sensor were addressed and a portable low-cost system with a high accuracy and repeatability was developed. The 3D printed packaging of the sensor prevented the damages previously caused by short-circuiting and ESD. The designed sensor consisted of four symmetrical sensing elements to eliminate the effects of local concentration of particles in inhomogeneous liquids on the sensor measurements. The calibration issue of the viscosity measurements caused by the indirect verification of sensor results is still under investigation.

## 4. Conclusions and Future Work

In this work, the concept of viscosity measurement of sputum samples for the early diagnosis of COPD using a dielectric sensor was investigated. Two prototypes of the dielectric sensors were designed and fabricated using the CMOS technology. The sensors measured the permittivity variation of the MUT which was correlated to their viscosity changes. The limitations of the first prototype were identified and addressed and a newer version of the sensor was developed. The sensor performance for detecting the viscosity of liquids was evaluated using controls and through the mixture characterization method. The sensor assessment results show that the RF sensor is capable of measuring the dielectric constant of liquids with an accuracy and a repeatability of 4.17% and 5.36%, respectively. Moreover, low amounts of noise and drift were observed during measurements, providing reliable results for long-term medical applications. In addition, the packaging of the sensor simplified the system handling during operation. The DC readout mechanism of the second prototype as well as its compact size and low power consumption made it a suitable technology for POC applications. Ease of cleaning, portability, low cost, and capability of rapid detection of viscosity are among the main novelties of this biosensor for clinical applications.

As a next step, the sensor will be calibrated using a commercialized viscometer and its performance will be evaluated during the characterization of non-Newtonian fluids. Considering the practical application of this sensor, providing absolute viscosity values is necessary for COPD diagnostics. In addition, the developed sensor will be incorporated into a complete hand-held device suitable for POC and IoT applications.

## Figures and Tables

**Table 1:** Dielectric constant of utilized materials at 30 GHz and 21 ${}^{\circ }$C [32,33,34,35].

Material	Dielectric Constant
Air	1
Isopropanol	3.08
Ethanol	4.51
Methanol	8.2
Acetone	15.4

**Table 2:** Results of the sensor verification experiments for methanol dielectric constant.

Methanol	Actual Value	Exp. 1	Exp. 2	Exp. 3
Dielectric Constant ($\varepsilon_{r}^{\prime }$)	8.2	8.82	8.14	8.65

**Table 3:** Results of the performance evaluation of the second generation of the dielectric sensor.

	Accuracy	Repeatability	Hysteresis	Drift	Noise
$\varepsilon_{r}^{\prime }$ /(mV)	4.17%	5.36%	0.014 (2 mV)	0.038 (5 mV)	0.006 (1 mV)
